# Insights into Cardiovascular Defects and Cardiac Epigenome in the Context of COVID-19

**DOI:** 10.3390/epigenomes6020013

**Published:** 2022-04-21

**Authors:** Shreya Sarkar, Rwik Sen

**Affiliations:** 1 New Brunswick Heart Centre, Saint John Regional Hospital, Saint John, NB E2L 4L2, Canada; shreya.sarkar@horizonnb.ca; 2 Active Motif, Inc., 1914 Palomar Oaks Way, Suite 150, Carlsbad, CA 92008, USA

**Keywords:** COVID-19, congenital heart defects, cardiovascular disease, epigenome, DNA methylation, non-coding RNA, ACE2, Ang1-7, Mas, physiology

## Abstract

Although few in number, studies on epigenome of the heart of COVID-19 patients show that epigenetic signatures such as DNA methylation are significantly altered, leading to changes in expression of several genes. It contributes to pathogenic cardiac phenotypes of COVID-19, e.g., low heart rate, myocardial edema, and myofibrillar disarray. DNA methylation studies reveal changes which likely contribute to cardiac disease through unknown mechanisms. The incidence of severe COVID-19 disease, including hospitalization, requiring respiratory support, morbidity, and mortality, is disproportionately higher in individuals with co-morbidities. This poses unprecedented strains on the global healthcare system. While their underlying conditions make patients more susceptible to severe COVID-19 disease, strained healthcare systems, lack of adequate support, or sedentary lifestyles from ongoing lockdowns have proved detrimental to their underlying health conditions, thus pushing them to severe risk of congenital heart disease (CHD) itself. Prophylactic vaccines against COVID-19 have ushered new hope for CHD. A common connection between COVID-19 and CHD is SARS-CoV-2’s host receptor ACE2, because ACE2 regulates and protects organs, including the heart, in various ways. ACE2 is a common therapeutic target against cardiovascular disease and COVID-19 which damages organs. Hence, this review explores the above regarding CHDs, cardiovascular damage, and cardiac epigenetics, in COVID-19 patients.

## 1. Introduction

The world is currently facing a once-in-a-century pathogen, responsible for the ongoing pandemic called COVID-19 or coronavirus disease 2019 [1]. COVID-19 is caused by the novel severe acute respiratory syndrome coronavirus 2 (SARS-CoV-2) [2]. First identified in the city of Wuhan in Hubei Province, China, in the middle of December 2019 [3], COVID-19 has now spread globally. According to the WHO (World Health Organization), as of 18 March 2022, there have been 465,147,337 confirmed cases of COVID-19, including 6,083,874 deaths globally. The European Centre for Disease Prevention and Controls reported 458,179,120 cases of COVID-19 (in accordance with the applied case definitions and testing strategies in the affected countries) and 6,058,022 deaths as of 18 March 2022. Predominantly a respiratory coronavirus, SARS-CoV-2 is primarily transmitted through respiratory droplets, with a median incubation period of 4–5 days, and ~98% of patients developing clinical symptoms within 11.5 days [4]. The SARS-CoV-2 infection cycle begins with the viral spike or S protein binding to the host cell surface receptor called ACE2 (Angiotensin-converting enzyme 2) which facilitates viral entry into the host cell. ACE2 is a pleiotropic mono-carboxy peptidase of the zinc metalloproteases family, present ubiquitously in plasma and tissues [5]. ACE2 is part of the protective ACE2-Ang-(1-7)-Mas receptor axis of the renin–angiotensin system (RAS) which counteracts the deleterious RAS axis called ACE-Ang II-AT1 [6,7,8,9,10]. Interestingly, ACE2 and its axis partners regulate the development and physiology of various organs, inflammation, immunity, ageing, disease, and detrimental effects of environmental factors such as nicotine [7,11,12,13]. However, unlike other related coronavirus-induced diseases, COVID-19 is unique in its ability to affect different organs and exhibit a broad spectrum of clinical manifestations including end-organ damage [14,15].

COVID-19 disproportionately affects the elderly population, with people above the age of 65 years representing ~80% of hospitalizations and having 23-fold higher risk of death than those below the age of 65 years [16]. Risk factors such as comorbidities (diabetes, obesity, cardiovascular diseases), combined with genetic difference between the age groups explains in part the reasons of vulnerability towards COVID-19 [17]. However, vulnerability is a relative and highly contextual term, and the current pandemic has pushed a large proportion of individuals within the vulnerable bracket, for example, due to loss of income or access to social support, thereby exposing them to a higher risk COVID-19 [18]. Children have an overall low risk to COVID-19 disease [19,20], with severe disease or mortality due to underlying comorbidities [21].

Considering the above, it is not surprising that the COVID-19 disease burden tends to overwhelm the healthcare system and induces massive disruption of essential healthcare services [22,23,24]. COVID-19 affects the cardiovascular system, and conversely, pre-existing cardiovascular conditions lead to worse outcomes, end-organ damage, and increased risk of mortality in patients with COVID-19 [25]. Therefore, COVID-19 infection in children with pre-existing congenital cardiac defects pose not only higher risks of worse outcomes due to COVID-19, but also lack of essential services for the treatment of pre-existing conditions.

Since epigenome-based therapies are an upcoming and promising field, it is necessary to explore their potential in our battle against an unfamiliar enemy when there is a lack of current preventative strategy. In this direction, few studies have investigated the epigenome upon SARS-CoV-2 infection to find several alterations in DNA methylation which is a major epigenetic signature, and some of these studies investigated the heart [26,27,28,29,30]. Consequently, altered DNA methylation has led to changes in the expression of several genes. Since epigenetic impacts to the transcriptome are connected to cardiac pathologies, it will be interesting to investigate the connections between the epigenomic changes in COVID-19 patients and their pathogenic cardiac phenotypes such as low heart rate, myocardial edema, myofibrillar disarray, and relationship to comorbidities such as heart failure.

In this direction, this review presents the current scenario of cardiovascular diseases and congenital heart defects associated with COVID-19 patients, and provides a developmental biology perspective of the roles of ACE2 and its axis members, because ACE2 is the host protein that is under focus in COVID-19 research. Interestingly, ACE2 and its axis members promote organ protection including cardioprotection and cerebroprotection, while end-organ damage is a major outcome of COVID-19. The review presents studies on cardiac epigenome upon SARS-CoV-2 infection, the roles of ACE2 in development and physiology leading to a focus on its roles in development and physiology of the heart and vasculature.

## 2. Altered Epigenomic Signature of DNA Methylation in Heart Impacts Transcriptome during SARS-CoV-2 Infection

COVID-19 alters epigenetic signatures such as DNA methylation and histone modifications, transcriptome and proteome which have been published as original research and reviews [31,32,33,34,35,36,37,38,39,40,41,42]. Among histone modifications, COVID-19 patients show elevated histone H3 citrullination which marks neutrophil extracellular traps (NETs) or NETosis, a phenomenon that is highly detected upon SARS-CoV-2 infection [40]. NETs are immune responses to infection hence the above observation establishes a direct link between histone modifications and immune response upon SARS-CoV-2 infection. Elevation in circulating histone-DNA complexes and histone H3 cleavage have been reported in severe COVID-19 cases [43,44]. Histone modifications for transcriptional activation such as H3K4me3 are reported in interferon response genes upon SARS-CoV-2 infection, and components of the SWI/SNF chromatin remodeling complex have been associated with SARS-CoV-2 infection cycle [45]. However, histone modifications are among the least explored domains in COVID-19 publications so far. Hence, further research on the underlying mechanisms of above inferences is needed. In the context of DNA methylation, the SARS-CoV-2 receptor in humans, *ACE2*, is reported to undergo altered DNA methylation in COVID-19 which can be potentially targeted for therapies [33,34,35]. 

Apart from histone modifications and DNA methylation, non-coding RNA (nc-RNA) is an important contributor of epigenetic changes. In COVID-19, several nc-RNA are upregulated or downregulated which render them as potential biomarkers of the disease [46,47,48,49,50,51,52]. Askari et al. reported that several long nc-RNAs and micro RNAs (mi-RNAs) are differentially expressed in COVID-19, some with specific observations in female versus male patients [46]. For example, comparison of female versus male COVID-19 ICU patients showed upregulation in *XIST* lncRNA, while downregulation in *TTTY14*, *TTTY10*, and *ZFY-AS1* lncRNAs [46]. They further identified several miRNAs with multiple binding sites on SARS-CoV-2 genome, e.g., miR-29b has five binding sites. Not only are nc-RNAs potential biomarkers, they are also important regulators. For example, miR-29a/c targets differentially expressed genes (DEGs) that are induced by COVID-19, while miR-125 and others target DEGs regulating immune response [46]. Interestingly, nc-RNAs have significant roles in cardiac biology, including miR-29s and miR-30b reported in this study, which have anti-fibrotic roles in the heart and correlate with ischemic heart disease, respectively [46]. Badimon et al. provides a comprehensive compilation of nc-RNAs, including miR-16-2-3p, lncRNA *NEAT1*, which are upregulated in COVID-19 patients, and lncRNA *DANCR*, miR-183-5p, etc., which are downregulated [47]. Such differentially expressed nc-RNAa can serve as potential biomarkers of COVID-19. Several studies on epigenetics of COVID-19 have highlighted the adverse effects of SARS-CoV-2 infection on the heart, such as cardiac failure, myocarditis, pericarditis, and myocardial infarction [27,53,54,55,56,57,58,59]. 

**Figure 1:**
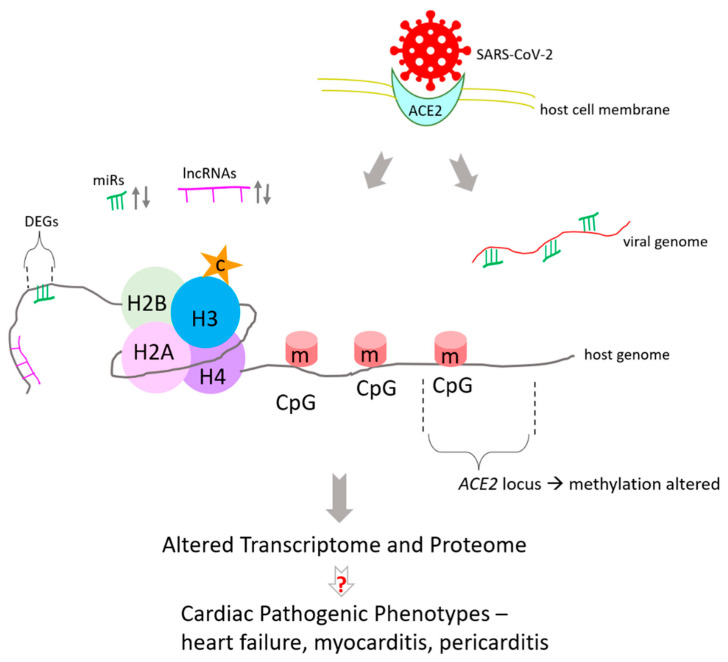
SARS-CoV-2 infection changes impacts the epigenome. DNA methylation is altered, including at host receptor *ACE2*. “m” on CpG islands represent DNA methylation. H2A, H2B, H3, and H4 are core histones which DNA wraps around. Histone H3 citrullination (c) is elevated in COVID-19. Several host micro RNAs (miRs) and long non-coding RNAs (lncRNAs) are differentially expressed (↑↓) in COVID-19. They impart epigenetic regulations including those at DEGs (differentially expressed genes) induced by COVID-19. Several miRNAs have multiple binding sites on SARS-CoV-2 genome. Epigenomic changes impact transcriptome and proteome. Molecular mechanisms behind epigenomic perturbations leading to organ damage and dysfunction in COVID-19 are unknown (indicated by “?”).

One of the few studies that investigated cardiac epigenome in COVID-19 was Li et al. [26]. They developed a mouse model of COVID-19 where wild-type 6-week-old mice were intraperitoneally injected with adeno associated virus serotype 9 (AAV-9) encoding the human ACE2 (hACE2) transgene, followed by injection of SARS-CoV-2 virus isolate after 2 weeks. The murine model developed systemic toxicity due to the infection and demonstrated cardiac pathogenic phenotypes that are common to humans. DNA methylation on cytosine residues is an important epigenetic signature that affects gene expression. CpG islands are regions in the genome containing 5′—Cytosine —phosphate —Guanine—3′, where methylation of cytosine epigenetically alters gene expression (Figure 1). Hence, methylation status of genes from heart tissue was analyzed 7 days after SARS-CoV-2 infection of murine models. The results showed that 172 sites in the heart were differentially methylated. 

Among several genes, two genes with multiple differentially methylated sites in heart were *Peg10* and *Ece1*. Upon SARS-CoV-2 infection, *Peg10* was found to be hypomethylated and subsequently its expression was upregulated. *Ece1* was hypermethylated upon infection and its expression was altered. Interestingly, *Peg10* regulates cell proliferation, binds viral transcription factors, while its loss of function is lethal to early stages of developing embryos [60,61,62], and loss of function of *Ece1* correlates with heart defects, edema, and autonomic dysfunction [63]. Further studies are needed to establish the molecular mechanisms behind cardiac epigenome perturbations and cardiac pathogenic phenotypes in COVID-19, but this study clearly indicates that altered DNA methylation in the heart is likely a contributing factor [26]. It also indicates towards a prenatal link between maternal SARS-CoV-2 infection and altered DNA methylation. Kgatle et al. [29] and Papakonstantinou et al. [30] have also discussed epigenome in the context of COVID-19 and heart.

DNA methylation of COVID-19 patients have also been studied by Castro de Moura et al. [27] and Balnis et al. [28] where blood samples were analyzed. Castro de Moura et al. obtained peripheral blood samples from 407 COVID-19 patients and controls, and found that 44 DNA methylation sites correlated with the clinical severity of COVID-19 [27]. Balnis et al. obtained DNA from frozen whole blood of 102 COVID-19 patients and controls, and reported that 77 regions of DNA were differentially methylated which predicted the severity of COVID-19 [28].

## 3. COVID-19 and ACE2

Congenital heart defects and cardiovascular disease make patients more susceptible to heart failure and pose a higher mortality risk as comorbidities in COVID-19 patients and significantly challenge treatment [64,65,66]. It is important to note that in addition to being targeted for COVID-19 therapies, ACE2 is proposed as a therapeutic target against heart failure, cardiotoxicity, and other cardiac disorders [67,68,69,70,71]. The association between COVID-19, ACE2, and cardiovascular diseases is significant because it has received focus in other studies too [72]. Hence, this review aims to extend the information resources and draw from both the developmental biology aspect of ACE2 and heart disease associated with COVID-19 to inform future studies about the common grounds between the two seemingly distinct yet related areas.

### ACE2/Ang1-7/Mas Receptor Axis Regulates Development, Physiology, and Organ Protection

**Figure 2:**
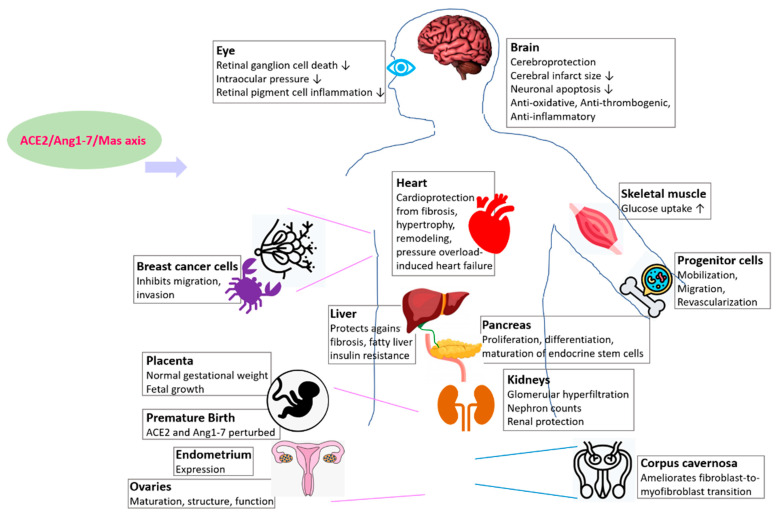
ACE2/Ang1-7/Mas axis plays a beneficial and protective role in regulating several organs and tissues. Features and roles of the axis in respective organs are mentioned in boxes. ↓ represents reduction.

The roles of ACE2/Ang1-7/Mas receptor axis has been reported in the development and physiology of several organs and tissue which are briefly summarized below (Figure 2, Table 1). As end-organ damage is an outcome of several COVID-19 patients and ACE2 regulates their development and physiology, as discussed below, the information can potentially help studies aiming to promote organ protection by modulating the ACE2/Ang1-7/Mas axis during disease, and studies that target ACE2 in therapeutic developments against both COVID-19 and heart disease.

In the context of heart, ACE2 is implicated in regulating cardiac structure and function, and the development of cardiovascular diseases [73,75,76,77]. Overexpression of ACE2 is beneficial for cardiac function, and opposes myocardial fibrosis and left ventricular hypertrophy [78,79,80,81]. Ang II promotes cardiovascular diseases whose progression is prevented upon inhibiting Ang II [82], hence one of the ways by which ACE2 benefits the heart is by hydrolyzing Ang II into Ang 1-7 [82,83]. Conversely, elevated intracardiac Ang II levels and absence of ACE2 leads to cardiac dysfunction and remodeling caused by pressure overload [84]. Further, ACE2 null mice show progressively reduced left ventricular contractile function, elevated AngII levels in plasma and tissue and low blood pressure in 6-months old male homozygous null mutant mice [85], and increased vulnerability to Ang II-mediated hypertension [86].

Events induced by Ang II such as cardiac hypertrophy, remodeling, and pressure overload-induced heart failure are attenuated by Ang1-7 [8,9,87]. Ang 1-7 renders cardioprotection and exerts beneficial effects on cardiac remodeling when co-infused with isoproterenol treatment in transgenic rats that elevates Ang1-7 levels [88,89]. Consistently, overexpression of cardiac-specific Ang1-7 in transgenic mice attenuates the effects of systemic infusion of Ang II in wild-type mice such as hypertrophy, fibrosis, hypertrophic, and profibrotic marker expression such as atrial and brain natriuretic peptides, transforming growth factor-β [90].

ACE2 regulates the prevention of progressive cardiac fibrosis in aging and cardiac pressure overload models [84,85,91,92]. The role of ACE2 in attenuating cardiac fibrosis and hypertrophy, left ventricular function, and remodeling following myocardial infarction, is further supported by other studies [76,93,94,95].

In addition to ACE2, Mas also prevents proliferation and fibrosis by regulating various matrix proteins because its deficiency results in elevation of fibronectin, collagen types I and III, and reduction in collagen IV in right ventricle and aortic valves in neonatal and adult mice [96]. Mas receptors are localized in cardiac myocytes, and they activate nitric oxide production for cardiovascular signaling [97]. 

Overall, Activation of ACE2/Ang(1-7)/Mas receptor axis counteracts vasoconstriction, hypertrophy, and fibrosis hence improves cardiac function, remodeling, and attenuates heart failure [75,98,99,100]. Based on their roles in cardioprotection, the ACE2/Ang(1-7)/Mas receptor axis is a potential therapeutic target against end-organ damage [7,101,102]. In this direction, a recent study on in 110 heart failure patients show increased Ang 1-7/Ang II ratio as independent and incremental predictor of reduced hospitalization time, better outcomes, and survival [103]. Further, AVE0991, an orally active Ang-(1-7) analog recapitulates cardioprotective roles of Ang1-7 [104]. As ACE inhibitors and blockers of angiotensin receptors are ineffective against coronary artery disease and hypertension, the effects of AVE0991 is clinically significant and promising [75]. Together, the clinical studies support the therapeutic targeting of ACE2/Ang(1-7)/Mas receptor as a promising approach towards cardioprotection and therapeutic developments against COVID-19.

## 4. Cardiovascular Damage during SARS-CoV-2 Infection

COVID-19 patients show acute myocardial injury and chronic cardiovascular damage, which are conditions whose risks are increased by CHDs. In order to protect against the above conditions during COVID-19 infection and treatment, the cardiovascular impact of SARS-CoV-2 infection needs to be well-studied. This is because COVID-19 treatment becomes extremely inconvenient and complicated if patients have additional conditions such as myocardial damage which often lead to death. To make matters worse, acute cardiac injury is more prevalent in COVID-19 patients with fatal outcomes, while patients presenting cardiovascular risk factors are at supposedly increased risk of death from COVID-19 [105,106,107,108,109]. In this direction, several interesting observations have been reported as presented below.

A study showed that 36 COVID-19 patients in the ICU have significantly elevated levels of myocardial injury biomarkers such as median creatine kinase myocardial band (CK-MB) and high-sensitivity troponin I (hs-cTnI) [110]. In COVID-19 patients, increased levels of hs-cTnlI correlated with a four-fold higher risk of fatality in patients despite COVID-19 treatment being adjusted for age and pre-existing cardiovascular disorders [105,111]. Patients reported heart palpitations and chest tightness, while many patients who died of COVID-19 had cardiac arrest during hospitalization [110,112]. In another study, COVID-19 patients in the ICU showed cardiovascular complications such as elevated blood pressure, elevated cardiac biomarker levels in serum, and abnormalities detected by electrocardiography and echocardiography [113]. In the past, infection of SARS-CoV, from which SARS-CoV-2 is derived, has been reported to cause chronic cardiovascular damage in 44% of 25 patients [114]. It has also been shown that systemic symptoms and severe pneumonia are higher in SARS-CoV-2 patients above 60 years of age, which can predictably worsen due to underlying cardiovascular conditions [115].

Several studies reporting COVID-19 fatalities and associated cardiovascular disease have presented that 15–70% of patients who had COVID-19-related deaths also had underlying cardiovascular disease [105,106,107,109]. This important observation calls for a focus on understanding whether any particular predisposition for COVID-19 patients with pre-existing cardiovascular disorders exists. This hypothesis is further supported by studies reporting probable connections among cardiovascular comorbidity and high severity of SARS-CoV-2 infection [106,116], and myocardial dysfunction in 20–30% of COVID-19 patients in ICU [108]. Moreover, fourteen different studies have reported the presence of a minimum of two cardiac biomarkers in COVID-19 under hospitalization [108]. 

Complications associated with blood coagulation factors have also been observed in COVID-19 patients. Abnormalities associated with higher risks of thromboembolism of both veins and arteries have been linked to COVID-19 patients [117,118]. Systemic thromboprophylaxis has been reported in COVID-19 patients among whom approximately 31% patients show thrombotic complications, where pulmonary embolism is projected as the primary cause of complications [119]. Other than pulmonary embolism, some of the other complications seen in COVID-19 patients include deep vein thrombosis, ischemic stroke, myocardial infarction, and peripheral arterial thromboembolism. Several other studies have reported frequent events of venous and arterial thrombosis in COVID-19 patients, where 27–69% cases are for peripheral venous thromboembolism, approximately 79% cases for deep vein thrombosis and pulmonary embolism [120,121,122]. One of these studies reported 31% cases of combined arterial and venous thrombosis in 184 patients at ICU having COVID-19 pneumonia although they show appropriate prophylactic anticoagulation [120]. 

In support of these observations, other studies have also reported a higher risk for venous thromboembolism during severe SARS-CoV-2 infection [123]. In this direction, a study on 143 COVID-19 patients presents lower extremity deep vein thrombosis in 46% patients, where these 46% patients show worse prognosis, increased cardiac injury, and mortality [118]. Further, autopsies have revealed pathological information implicating elevated risk of thrombosis in COVID-19 patients such as angiogenesis and severe endothelial injury angiogenesis [124,125].

At the molecular level, studies have identified hemostasis-associated abnormalities in COVID-19 patients that include elevated levels of D-dimer and fibrin degradation entities, lengthened thrombin and prothrombin durations and international normalized ratio, reduced activated partial thromboplastin duration, positive antiphospholipid syndrome related antibodies, and thrombocytopenia coupled with traditional comorbidities [106,126,127,128,129].

The cytokine storm in COVID-19 patients causes extreme inflammation stress which likely leads to rapid inflammation in vascular tissue that leads to atherosclerosis, cardiac arrhythmia, and myocarditis [116]. It is speculated that the intense inflammation during COVID-19 makes patients prone to intravascular thrombosis which elevates levels of blood clotting factors. A predisposition of COVID-19 patients to thrombosis likely results from direct and indirect impacts of SARS-CoV-2 and processes associated with its infection such as severe inflammation, critical illness, and hypoxia [126]. The complications are exacerbated in combination with immobilization and pre-existing comorbidities which lead to venous thromboembolism [130]. Studies are ongoing to identify the detailed mechanisms that link COVID-19 and disrupted blood coagulation. Nonetheless, the collective observations from the above reports coupled with the fact that COVID-19 patients show a high frequency of ischemic stroke [131], indicate that vascular thrombosis is an integral part of COVID-19 [108].

## 5. Congenital Heart Disease

COVID-19 treatment is challenged in patients with congenital heart disease (CHD) because CHDs are considered comorbidities for COVID-19 that increase mortality risk in patients. Hence, detailed connections between COVID-19 and CHDs need to be investigated to develop personalized therapies for COVID-19 patients with CHDs. In this direction, significant focus is required on CHDs. 

CHDs are the most common congenital defect in newborn babies affecting 0.8% of live births, and includes abnormalities in the heart structure or great vessels which occur during the development of the fetus at pregnancy [132,133]. It is estimated that 1 in every 100 children has defects in the heart due to underlying genetic or chromosomal anomalies [132], 40% of which are diagnosed in the first year of life [134]. However, the true prevalence might be significantly higher. CHD is also the most prevalent cause of infant deaths from birth defects [133]. Risk factors include alcohol, drug and medicinal abuse during pregnancy, viral infections during the first trimester, maternal diabetes, obesity and other complications, and family history [132,133]. Outcomes vary with socio-demographic index, highlighting the important for introducing policies to address these global inequalities for optimum amelioration of the disease [135].

**Figure 3:**
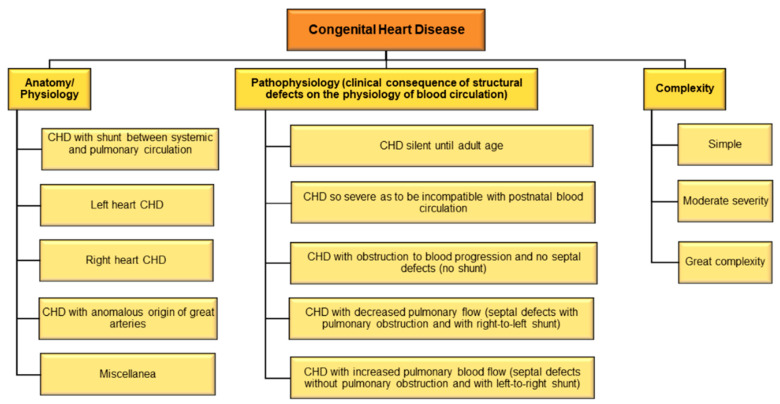
Classification of CHD.

There are different types of CHD and consequently, different methods of disease classification (Figure 3). Considering the underlying anatomy and pathophysiology, CHD may be classified as (1) CHD with shunt between systemic and pulmonary circulation, (2) left heart CHD, (3) right heart CHD, (4) CHD with anomalous origin of great arteries, and (5) miscellanea [136]. Based on pathophysiology using clinical consequence of structural defects on the physiology of blood circulation, CHD may be classified as (1) CHD with increased pulmonary blood flow (septal defects without pulmonary obstruction and with left-to-right shunt); (2) CHD with decreased pulmonary flow (septal defects with pulmonary obstruction and with right-to-left shunt); (3) CHD with obstruction to blood progression and no septal defects (no shunt); (4) CHD so severe as to be incompatible with postnatal blood circulation; and (5) CHD silent until adult age [137]. 

A useful and rapid method of classification categorizes CHD into CHD of great complexity, CHD of moderate severity, and simple CHD [133]. A recent study also classifies adult CHD anatomic and physiological parameters to predict 15-year cardiac mortality [138]. The International Paediatric and Congenital Cardiac Code (IPCCC) and the Eleventh Iteration of the International Classification of Diseases (ICD-11) have devised standard codes for classifying CHD [139]. An older study had shown that ICD codes may lead to substantial misclassification of CHD [140]; however, recent studies to validate if newer classifications have been deemed effective are warranted.

## 6. Diagnosis of CHD

**Figure 4:**
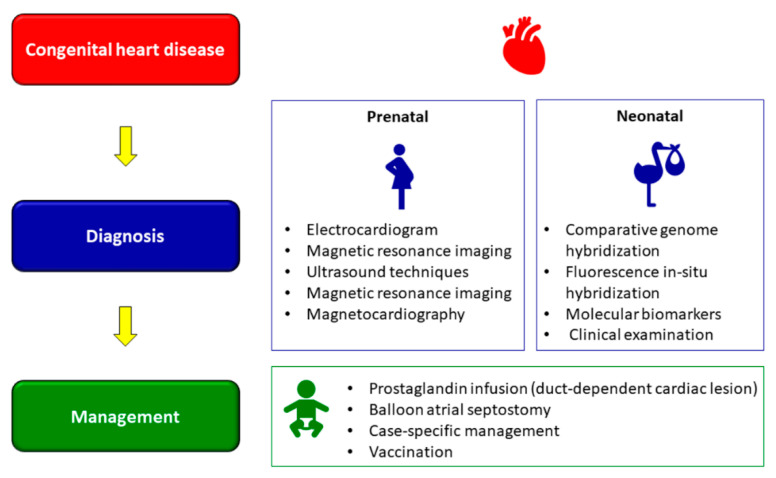
Diagnosis and management of CHDs.

Recent advances in medical technology and diagnostics have facilitated in early detection of CDHs (Figure 4), though mortality, albeit decreasing, still remains unacceptably high [141]. Prenatal diagnosis using two-dimensional fetal echocardiography (ECG) was the conventional method of prenatal diagnosis of CHD; however, this has been replaced by contemporary three-dimensional and four-dimensional ECG over the past decade [141]. Other methods of detection include advanced ultrasound techniques, fetal magnetic resonance imaging, and fetal magnetocardiography [142]. Many studies have shown the accuracy of prenatal diagnosis using ECG [143,144,145,146,147]. However, all these methods have inherent limitations as to what can and cannot be detected and interpretated, not limited to imaging and anatomy, thereby necessitating novel methods of diagnosis [141,148]. 

A recent research study showed that magnetic resonance imaging, in combination with gene analysis using array comparative genome hybridization analysis and fluorescence in situ hybridization could be a more effective diagnostic method for CDHs [149]. Similarly, molecular biomarkers too have shown considerable promise for use in prenatal diagnosis of CHD [150,151,152]. In addition to the above, detection of CHD neonates include clinical examinations, such as for congestive heart failure, a rhythm disturbance or heart murmur, cyanosis, and measurement of transcutaneous oxygen saturation [153,154]. Neonatal diagnosis has shown to be successful in detecting CHD and predicting outcomes [155]. Despite of all the advances in early detection of CHD, what remains unavoidable is the impact CHD diagnosis on parents, not limited to stress, uncertainty and other psychological turmoil [156,157,158], and the need for mental/social support to address such concerns [159,160].

## 7. Management of CHD

Management of CHD often begins following a prenatal diagnosis (Figure 4). As some newborns require urgent care immediately after delivery, planning becomes an essential consideration for such cases. Delivery close to a pediatric cardiac centre, in the presence of a specialized cardiac team or with arrangements for urgent transportation of the newborn if required helps improve outcomes of neonates with a prenatal CHD diagnosis [161]. Management of CHD in neonates is an elaborate, complex process and highly contextual, the elaboration of which is beyond the scope of this review. Depending on the condition, some newborns with ductal-dependent cardiac lesions are given prostaglandin infusions [162]. Prostaglandin E1 (PGE1) helps in maintaining ductal patency, which in turn promotes mixing of pulmonary and systemic blood flow or improve pulmonary or systemic circulations and is hence administered prior to balloon atrial septostomy or surgery [163].

One of the oldest interventions to palliate certain types of CHD is balloon atrial septostomy, described almost half a century ago [164,165]. The objective of this intervention is to widen a restrictive atrial communication, thereby enhance atrial mixing, decompression of the left atrium and augmentation of the cardiac output in right-side obstruction lesions [164,165]. Initial surgical interventions in patients with certain types of CHD is mostly palliative rather than reparative, and these patients often require subsequent surgeries throughout their lifetimes [166]. Case specific medical management is also used in conjunction with invasive interventions [166] Details of the different CHD and the types of management required have been previously elaborated [166,167]. 

In addition to management of symptoms, children with CHD are at a higher risk of severe illness and hospitalization due the respiratory tract infections [168]. Of special mention are human respiratory syncytial virus (HRSV) and influenza virus, both of which lead to cardiopulmonary compromise and adverse outcomes including acute kidney failure, pneumonia, morbidity, and mortality [169,170,171]. Routine vaccination with special focus on the medical condition of these children is a proven powerful and dynamic weapon vital to their quality of life and long-term survival [172,173]. Infectious disease prevention is a collaborative approach, wherein healthcare providers need to be constantly updated regarding latest news, recommendations, and warnings regarding vaccines, while families/caregivers need to be vigilant regarding nutritional status, vaccinations, and prevention of winter illness in children with CHD [173,174]. 

## 8. CHD during the COVID-19 Pandemic

The COVID-19 pandemic has put the global healthcare system on the edge and has severely affected the management of different acute and chronic diseases including CHD. COVID-19 patients have an increased risk of heart failure (Freaney 33,001,179), while patients with low LVEF (left ventricular ejection fraction), which is an indicator of heart failure, are associated with increased susceptibility to COVID-19 (33,205,916 Matsushita, 32,509,415 Sinkey). Cardiovascular abnormalities are reported in case studies on multiple COVID-19 patients (Grillet 32,324,103, Maham 32,437,313), and the list of complications includes tachycardia, non-obstructive coronary artery disease, and non-ischemic cardiac myopathy (Fried 32,243,205). A study on neuroimaging on 725 hospitalized COVID-19 patients revealed some cases on cardioembolism where the heart propels undesired entities to brain circulation resulting in stroke, as well as the presence of other cardiovascular abnormalities (Maham 32,437,313). Hence, the cardiovascular implications also extend to neurological dysfunctions. CHDs render patients more susceptible to heart failure and COVID-19 affects the cardiovascular system with symptoms often similar to those of CHDs. Therefore, distinguishing between the two might prove challenging [175]. Many children with COVID-19 are asymptomatic or have minimum symptoms; therefore, the magnitude of children with CHD and infected with COVID-19 is difficult to assess, as presented by Lewis et al. [176]. This study focused on COVID-19’s impact and predictors in patients with CHDs, but the number of symptomatic COVID-19 patients in this study was low. 

Nonetheless, the study has significant clinical implications including the identification that if these patients have a genetic syndrome and if they are adults at an advanced stage of physiology, then their risk for moderate to severe COVID-19 infection is maximum [176]. The study reports that out of 53 COVID-19 patients with CHDs, 52 were symptomatic. CHDs in these patients include 16 cases or 30% patients showing tetralogy of Fallot or pulmonary stenosis, 10 cases of 19% patients showing single ventricle physiology status post Fontan palliation, and 6 cases or 11% patients showing shunting defects. Further, seven cases for each of the three conditions were seen: i. congenital valve abnormality, ii. atrioventricular canal defects, and iii. anomalous left and right coronary arteries, coarctation of the aorta, double-chamber right ventricle, pulmonary atresia, D-transposition, and congenitally corrected transposition of great arteries [176]. Lewis et al. have presented detailed analyses of these patients, where a key observation highlights that the duration of COVID-19 symptoms is longer in patients with ventricular dysfunction.

Moreover, although children are at low risk of mortality from COVID-19 and there are insufficient data on COVID-19 in children, experience with previous viral diseases, including influenza and respiratory syncytial virus, makes it reasonable to extrapolate that COVID-19 would severely affect children with CHD [175]. Furthermore, CHD is associated with a multitude of comorbidities and health complications, thereby heightening the risk of COVID-19 [177]. Infants and young children with CHD appear to be more susceptible to COVID-19 than older children with CHD, as shown by a handful of case studies [178]. Another study describes a few cases of children with pre-existing CHD and infected with COVID-19. The children had adverse outcomes mostly due to exacerbation of their pre-existing conditions or missed COVID-19 symptoms due to their similarity with CHD symptoms [179].

Urgent invasive interventions, such as cardiac surgery to ameliorate CHD in newborns and infants, have proven especially challenging during the pandemic. The pandemic has stretched the healthcare infrastructure, and challenges in the face of CHD include looming resource scarcities of equipment, personnel, and blood, as well as infection risks in patients, caregivers, family members, and healthcare providers [180]. Wearing of masks and physical distancing for children is often an obstacle, and children mostly present as asymptomatic carries of COVID-19. Special attention to testing/screening of the patients and their families and routine use of personal protective equipment for healthcare workers is pertinent to limit spread COVID-19 in these vulnerable children with pre-existing CHDs [181] A Canadian study has brought to light the health management of children with CHD. These children are at risk of both COVID-19 disease and secondary cardiovascular outcomes due to limitations in physical activities imposed to control the pandemic. The study has shown a drop in their physical activity during the pandemic, thereby highlighting future challenges for both the patients and the healthcare system in managing CHD [182]. 

## 9. Vaccination for Improving Outcomes in CHD

For the last few centuries, vaccination, especially prophylactic vaccination, undoubtedly remains the most effective means of preventing infectious diseases [183]. Not surprisingly, global vaccination as a prevention of COVID-19 has opened new avenues to COVID-19 prevention/management, yet poses many questions and challenges. Two mRNA-based vaccines, independently developed by Pfizer/BioNTech and Moderna [184,185], have already been authorized, pre-ordered, and are in the process of being administered in several countries including the US, Canada, and EU [186]. Each country has prioritized certain individuals for receiving COVID-19 vaccination including those at high risk of illness/mortality, those at high risk of exposure to the disease including essential workers, and those needing special benefits such as minority populations [187]. Needless to say, CHD patients will be prioritized for COVID-19 vaccination, as these patients are at a high risk of developing COVID-19 [177]. This assumption is an extrapolation from the standard of care for CDH, where vaccination from other respiratory viruses such as influenza virus and RSV have proven effective as previously elaborated. Therefore, COVID-19 vaccination ushers in new hope for children suffering doubly from CHD and an ongoing pandemic, both with no end in sight. 

However, what is of concern is the equitability of global vaccine purchase and mobilization. Richer countries, accounting for only 13% of the global population, have already secured vaccine doses, leaving dwindling short-term supplies for low- and middle-income countries [186]. Variations in vaccine pricing and ultra-low handling and storage temperatures are other obstacles in equitable distribution of COVID-19 vaccines globally, with developing countries lacking sufficient infrastructure for the same. As the incidence of CHD in developing countries is relatively higher compared to developed countries [188], most children with CHD and their families/caregivers/healthcare system face unforeseen uncertainties and unprecedented challenges in CDH management in the era of the current pandemic.

## 10. Conclusions

**Figure 5:**
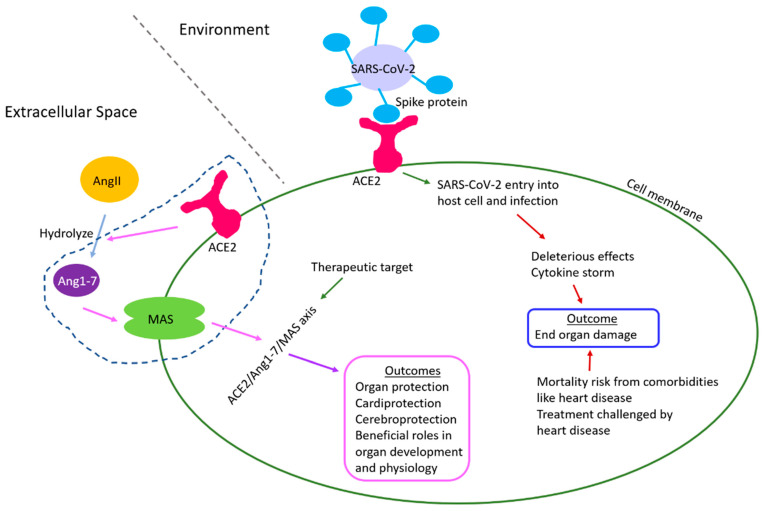
ACE2 is the host receptor for SARS-CoV-2 infection, and it is also a part of ACE2/Ang1-7/Mas axis. While COVID-19 causes cytokine storm leading to organ damage which is exacerbated in patients with heart disease, ACE2/Ang1-7/Mas axis renders organ protection and other beneficial roles in development and physiology. ACE2 is a therapeutic target for both COVID-19 and heart failure.

Heart diseases, including CHDs and cardiovascular disorders, are considered comorbidities that render a major mortality risk to COVID-19 patients and make the treatment procedure extremely challenging. CHDs and cardiovascular disorders increase the susceptibility to heart failure which is much increased in patients of COVID-19. Epigenetic perturbations through DNA methylation, histone modification, nc-RNA, etc., with alterations in transcriptome and proteome are reported in COVID-19 patients. The host receptor for SARS-CoV-2 infection, ACE2, regulates organ development, physiology, and protection including the heart, while COVID-19 often leads to multi-organ damage and failure, which has negatively impacted organ donation and transplants [189]. In this direction, this review presents information on the epigenetic implications of COVID-19 involving *ACE2* and the heart, the physiological roles of ACE2, and the current scenario of congenital heart defects and cardiovascular damage in COVID-19 patients, to draw from the above areas for informing future studies that address those questions (Figure 5). ACE2 is part of the ACE2/Ang1-7/Mas axis of RAS, and this axis is mainly known for beneficial effects on development, physiology, and protection of several organs as presented in this review. 

Although the role of ACE2 in cardiovascular disease etiology is not very well understood, ACE2/Ang1-7/Mas is strongly implicated in attenuating cardiac fibrosis, hypertrophy, and remodeling. Consequently, this axis is proposed as a therapeutic target against heart failure, ischemia, and other diseases as discussed earlier. In recent years, CRISPR (clustered regularly interspaced short palindromic repeats) has been successfully implemented in various applications including the editing of epigenetic factors [190,191] and as a potential option to address heart disease [192]. Based on the success of CRISPR, it is not surprising that CRISPR has been used to detect SARS-CoV-2 [193,194] and the advantages of CRISPR in various aspects of COVID-19 treatment are being considered [195,196]. As CRISPR is already established in the editing of epigenetic factors and cardiac research, it is only a matter of time before CRISPR is developed as a significant tool against COVID-19-related cardiac and epigenetic complications.

## Figures and Tables

**Table 1:** Organs and tissues as reported by studies whose development and physiology are regulated by ACE2/Ang1-7/Mas receptor axis.

Organ	Model Organism of Study	References
Kidney	Mouse	[73]
Liver	Mouse, rat	[74]
Pancreas	Mouse embryonic explant culture, peripheral blood CD34+ cells	[47,48,49,50]
Brain	Mouse	[51,52,53]
Eye	Rat, mouse, human	[54,55,56,57]
Placenta	Mouse	[58]
Skeletal muscle	Rat	[66,67]
Ovary	Human	[68,69]
Endometrium	Human	[70]
Corpus cavernosa	Rat	[71]
Breast (cancer)	Human	[72]
Hematopoietic and vasculogenic progenitor cells	Human, rodent	[73]
